# The Overworked Mind
*On the Supposed Increase of Insanity. By Edward Jarvis, M.D., of Dorchester, Mass. Reprinted from "The American Journal of Insanity."Life of Sir Walter Scott. By Mr. Lockhart.Remains of H. K. White.Memoir of Samuel L. Blanchard. By Sir Edward Bulwer Lytton, Bart., M.P.


**Published:** 1852-07-01

**Authors:** 


### THE JOURNAL

OF

### PSYCHOLOGICAL MEDICINE

AND

### MENTAL PATHOLOGY.

---

JULY 1, 1852.

---

### ART. I.—THE OVERWORKED MIND.*

THE wider spread of literature and science through society, the greater extent of commercial enterprise which has resulted from recent legislative and social changes, the larger amount of political action, whether municipal or parliamentary, accorded to the people, and the deep interest excited by free religious discussion, are causes, sufficiently obvious, for a much higher degree of mental activity than has hitherto characterized the two free nations of the world, the United Kingdom and the United States. Concurrently with this increase of mental activity, there has been a diminution of that amount of corporeal labour which is absolutely necessary to maintain a just balance between the spiritual essence and its organ. The merchant is confined to his counting-house; the student is tempted to push his sedentary habits into the hours required for repose; the politician undergoes every form of mental strain. But, in proportion as the cerebral system is worked, the muscular system is inactive, and at last mental labour is preferred to corporeal exertion, so that the man of thought becomes a mere lounger, incapable of any prolonged bodily effort. It is not surprising that, under these circumstances, there are manifested the phenomena of the OVERWORKED MIND.

Modern psychologists have not failed to notice the results of this

* On the Supposed Increase of Insanity. By Edward Jarvis, M.D., of Dorchester, Mass. Reprinted from "The American Journal of Insanity."
Life of Sir Walter Scott. By Mr. Lockhart.
Remains of H. K. White.
Memoir of Samuel L. Blanchard. By Sir Edward Bulwer Lytton, Bart., M.P.

excessive intellectual activity on the mental powers, particularly with reference to the increase in the numbers of the insane. Amongst the most recent and the most eloquent is Dr. E. Jarvis, of Dorchester, Massachusetts, whose essay "On the Supposed Increase of Insanity," reprinted from the "American Journal of Insanity," is before us. After detailing the statistics of insanity in various nations, and passing in review the prevalence, more or less marked, of the exciting and predisposing causes, Dr. Jarvis observes that "the causes connected with mental labour, in its manifold applications, have increased and are increasing continually. . . . . The improvements in the education of children and youth have increased their mental labours, and imposed more burdens upon their brains, in the present than in the preceding ages. The proportion of children who are taught in schools increases every year in the United States, and in most civilized nations. There are more and more of those whose love of knowledge, whose sense of duty, whose desire of gratifying friends, and whose ambition, impel them to make their utmost exertions to become good scholars. Thus they task their minds unduly, and sometimes exhaust their cerebral energies, and leave their brains a prey to other causes which may derange them afterwards. The new sciences which have been lately discovered, or the old sciences that were formerly confined to the learned, but are now simplified and popularized, and offered to the young as a part of their education, multiply the subjects of study, and increase the mental labour of almost all in schools."

This more widely extended education, as well with reference to the number of subjects for study, as of students, has a large influence on the adult mind. Men, and classes of men, Dr. Jarvis remarks, such as in the last century would have thought of nothing but how they should obtain their bread, are now induced to study subjects, and pursue sciences, and burden their brains with great, and sometimes with excessive, labour. New fields of investigation have been laid open within the last hundred, and especially within the last fifty, years. New inducements are offered, so that a greater variety of tastes is invited to their peculiar feasts of knowledge. Many persons now study phrenology, metaphysics, mathematics, physiology, chemistry, botany, and other branches of natural history, to say nothing of mesmerism, biology, &c. In this multiplication of students and of subjects for study, it is not surprising that some sink under the difficulties with which their weak judgments or enervated mental faculties are unable to grapple. Dr. Jarvis also refers, with great justice, to those public moral questions, which now more than formerly interest men's minds; as diet, temperance, public hygiene, &c.; all of which impose much mental labour on minds but imperfectly trained to endure it.*

Increased insanity is not, however, the only result of this excessive cerebral activity. It exercises an important influence on individuals of great social power, as writers, or statesmen, and upon the general mass of individuals in society. As to the former, the individuals themselves are the greatest sufferers; as to the latter, society. It cannot be doubted, we think, that if any agents, operating generally, so modify the corporeal organization, and the modes of mental action of large numbers of individuals (in virtue of their *general* operation) become imperfect, irregular, and unhealthy, we shall have the results displayed on a large scale in national eccentricities, and bizarre, peculiar, and unusual modes of thought and action, in sections and groups of individuals. And this proposition being granted, it further follows, that neither the political economist nor the philosophical statesman can be indifferent to the social condition of the people, as regards their intellectual development, and their too great or irregular exercise of the material organ of the mind. The psychologist and theologian will also enter upon the consideration of this matter with special interest, if he be thereby enabled to explain the deviations from a sound judgment on men and things, and the obliquity of moral vision, displayed by men of no obscure or unimportant position in the world of letters and philosophy. Superstitious and unhesitating credulity, and a strange cunning, have not been manifested in the lower or lowest classes exclusively. The national universities and the three learned professions have sent forth men who have adopted and strenuously promulgated dogmas so extraordinary, so irrational, and so utterly unfounded on fact, that their conduct has been referred to a form of monomania, or to wilful falsehood adopted for interested purposes. Yet probably the causes are really more on the surface, and are simply to be found in the corporeal results of an imperfect mental hygiene.

* "Marsilius Ficinus," Burton observes, "gives many reasons why students *dote* more than others: the first is their negligence. Other men look to their tools: a painter will wash his pencils; a smith will look to his hammer, anvil, forge; an husbandman will mend his plough-irons, and grind his hatchet if it be dull; a falconer or huntsman will have an especial care of his hawks, hounds, horses, dogs, &c.; a musician will string and unstring his lute, &c.; only scholars neglect that instrument (their brain and spirits, I mean) which they daily use, and by which they range over all the world, which by much study is consumed. The second is contemplation, which dries the brain and extinguishes natural heat; for, whilst the spirits are intent to meditation alone in the head, the stomach and liver are left destitute, and thence come thick and black blood from crudities, and for want of exercise the superfluous vapours cannot exhale."

The present social condition of civilized European nations has had its counterpart in all ages, and therefore presents numerous *historical* points of interest. A very cursory consideration of it from this point of view shows that the causes are complex, although obvious. These we have not at present, however, to investigate; as we propose on this occasion rather to consider the pathology and cure of the *overworked mind* in individuals. We cannot, however, pass over a remarkable illustration of the general fact we have stated, presented to us in the quaintly erudite pages of Burton. In his introduction to the "Anatomy of Melancholy," under the title of "Democritus to the Reader," Burton quotes at length from the Hippocratic writings the description of the visit of Hippocrates to Democritus, at the instance of the citizens of Abdera, who thought their philosopher and benefactor had become insane. Burton adds what fits to the present time: "Never so much cause of laughter as now; never so many fools and madmen. 'Tis not one Democritus will serve turn to laugh in these days; we have now need of a *Democritus to laugh at Democritus*, one jester to flout at another, one fool to flear at another—a great stentorian Democritus, as big as that Rhodian colossus; for now, as Salisburiensis said in his time, *totus mundus histrionem agit*—the whole world plays the fool: we have a new theatre, a new scene, a new comedy of errours, a new company of personate actors. . . . He that was a marriner to-day, is an apothecary to-morrow, a smith one while, a philosopher another, *in his volupiæ ludis*—a king now with his crown, robes, scepter, attendants, by and by drove a loaded asse before him like a carter; and if Democritus were alive now he should see strange alterations, a new company of counterfeit vizards, whiffers, Cumane asses, maskers, mummers, painted puppets, outsides, phantastick shadows, guls, monsters, giddy-heads, butterflies. . . . .

'———— ubique invenies

Stultos avaros, sycophantas prodigos.' "

Amongst the causes which operate most influentially in exciting these social aberrations, one of the most potent is, undoubtedly, the over-stimulated, over-worked, irregularly developed mind. It is a law of nature that health, ease, and order shall spring from labour, or from due use of the organs according to their appointed functions. This is universal. The "primal curse" is thus converted into a blessing. In all creation the due and regular performance of the allotted duties is rewarded by pleasing sensations, strength, and beauty, the undue and irregular, by pain, feebleness, deformity. This law holds good of the psychal as well as the physical, of the moral as well as the material.

"Through much tribulation ye shall inherit the kingdom," is a profound truth, whether that empire be corporeal power and beauty, or mental power and virtue. Here labour, however, is not thus rewarded. It must be well-directed, in harmony with the needs and powers of the individual—general, as regards the use of the organs, and not partial. Excessive labour in one exclusive direction produces corporeal deformity and mental obliquity. Just as the nursery-maid becomes the subject of spinal curvature and deformity, from the exclusive use of the right arm in carrying her precious burden, so the man of thought, who directs the energies of his powerful intellect to one subject or class of subjects, becomes mentally deformed. His judgment becomes one-sided, to use an expressive Germanism, or even imbecile, his manners bizarre, his conduct eccentric. It is thus that the eccentricities of men of genius are manifested, even to a proverb.

The evils of excessive study generally, and not simply in one exclusive direction, manifest themselves in morbid conditions of the organ of thought, which, reacting on the mind itself, disorder its manifestations. Hence, it has often been observed how narrow the bounds are between great genius and madness; how frequently the organ breaks down under the strain to which it is subjected. Hence it is that many intellectual suns have arisen in brightness, and set in clouds and darkness; have illumined the world by their morning or mid-day glory, and then have been for ever eclipsed by suicide, insanity, or idiocy :—

> "From MARLBOROUGH'S eyes the tears of dotage flow,
> And SWIFT becomes a driveller and a show."

Intermediately between the states of perfect vigour and complete disorganization, there are various phases of mental disorder, more distressing, perhaps, to the subject than even total extinction. No man feels more acutely than the man of letters, or the subject of prolonged intellectual labour, that state of mind in which every effort of thought is wearisome, and every object of thought is seen through a medium of gloom, anxiety, and dread. To such, existence is really a burden too heavy to be borne, and the endurance of life, under these circumstances, is probably as heroic an effort of fortitude as the endurance of a cruel martyrdom. The biographies of distinguished authors contain many touching instances of this kind.

Another result of mental toil is seen, not in the disorganization of the fibre of the brain so much as in the wearing out of the vascular system. Every effort of thought is accompanied by an expenditure of living material. The supply of this material is through the blood; hence the blood is sent in greater quantity to the brain in thought, and when the increased demand is constant, an increase in the vascular capacity of the brain becomes necessary, and is provided by the adaptive re-action of the organism. During the earlier periods of life this development of the blood-vessels only ministers to the vigour of the intellectual action; but when the decline of life commences, and the wear and tear of previous years shows itself, the increased vascularity is a source of danger, and lays the foundation for those diseases which depend upon congestion of the brain. Hence it is that apoplexy and palsy so frequently terminate the lives of great thinkers and writers. Hence, also, the proclivity of the literary and intellectual class to suffer fatally from those fevers and other diseases which attack the brain in preference to less important organs; and hence the distressing, sudden, and premature deaths of men of genius from causes and diseases apparently trivial. In some individuals, particularly those with co-existent disease of the heart and lungs, the vascular system gives way at once, and inflammation or apoplexy, epelipsy or acute mania, supervenes. The prime ministers of Austria and Prussia, during the recent revolutionary period, both succumbed to the overstrain of their material organ. Count Brandenburgh, of Prussia, died of inflammation of the brain after only a very short illness; Prince Schwartzenburgh of Austria perished in a moment, of apoplexy.

These various modifications of the mental condition are by no means the absolutely necessary results of mental labour. In the greater majority of studious men there already exists a predisposition to cerebral diseases, or else these are or have been present. This is manifested in various ways. In Scott and Byron the deformity of the foot and leg (talipes) of which they were the subject, indicated that a nervous attack occurred during intra-uterine life, of a paralytic or spasmodic character. Such an occurrence is apt to be accompanied by modifications of the mental characteristics: in some instances, by downright idiocy—this when the spasmodic attack has been severe and the deformity great; in others, by eccentricity, impetuosity of temper, waywardness, genius—and this when there is only a slight deformity, as a slight squint, twist of the foot, &c. Byron had, as a child, a temper sullenly passionate. In his case, the proclivity to irregular action of the nervous system and the peculiarity of temper were derived from his parents. His paternal ancestors were remarkable for their eccentricities, irregular passions, and daring recklessness; and his mother was liable to ungovernable outbursts of temper and feeling. With such parentage, and so constituted, it is not remarkable that Byron fell so early. It is not without a feeling of melancholy that we have perused Moore's account of his last moments; for the gifted biographer himself became subsequently the victim of his ardour, and his own glorious faculties were extinguished by mental, though not corporeal, death. Writing of Byron, he states: "The capricious course which he at all times pursued respecting diet—his long fastings—his expedients for the allayment of hunger—his occasional excesses in the most unwholesome food—and, during the latter part of his residence in Italy, his indulgence in the use of spirituous beverages—all these could not be otherwise than hurtful and undermining to his health. . . . . . When to all this we add the wasteful wear of spirits and strength from the slow corrosion of sensibility, the warfare of the passions, and the workings of a mind that allowed itself no Sabbath, it is not to be wondered at that the vital principle in him should so soon have burnt out, or that, at the age of thirty-three, he should have had—as he himself drearily expresses it—'an old feel.' To feed the flame, the all-absorbing flame, of his genius, the whole powers of his nature, physical as well as moral, were sacrificed—to present the grand and costly conflagration to the world's eyes, in which,

> 'Glittering like a palace set on fire,

> His glory, while it shone, but ruined him!'"

The fever of which Byron died displayed its fatal effects principally on the cerebrum. Whether the copious bleeding which was practised for his cure was judicious or not, we do not pretend to decide. We can affirm generally, however, that men and women so constituted seldom bear bleeding. The fate of the lamented Malibran comes to our remembrance, as we record Byron's protest against the depletion which was practised in his case. Referring to the opinion, as expressed by Dr. Reid in his essays, to the effect, "that less slaughter is effected by the lance than the lancet," he observed, "Who is nervous, if I am not? And do not those other words of his, too, apply to my case, where he says, that drawing blood from a nervous patient is like loosening the cords of a musical instrument whose tones already fail for want of sufficient tension? Even before this illness, you yourself know how weak and irritable I had become; and bleeding, by increasing this state, will inevitably kill me." We believe it is now thoroughly established amongst all judicious practitioners, that patients who have great cerebral activity, not only do not bear bleeding well, but have their lives endangered by loss of blood. We could refer to warning examples, if it were not a painful and invidious task to select them. We can assert with great certainty, however, that the *pabulum vitæ* must not be rashly withdrawn from the *overworked mind.*

Perhaps there is no more touching and instructive psychological history than that which details the phenomena of mental decadence, and bodily decline, amidst which the hand of the mighty magician of the north,

"Who rolled back the current of time,"

drooped—at last in helpless paralysis. In this mournful history (which, as detailed by Lockhart, we can never peruse without some wellings of emotion), there is chronicled the special physiology and pathology of the overworked mind. It is the history of a "case,"—too common, alas!—not to be neglected by those who now mount as upon the wings of eagles. At a time when pecuniary difficulties added to his mental labours, Sir Walter had to tug at the literary oar, and paid the first "penalty of his unparalleled toils" on the 15th February, 1830, when he had a slight apoplectic attack, more than two years and a half before his death. Mr. Lockhart justly remarks,—"When we recollect that both his father and elder brother died of paralysis, and consider the terrible violences of agitation and exertion to which Sir Walter had been subjected during the four preceding years, the only wonder is, that this blow (which had, I suspect, several distinct harbingers) was deferred so long; there can be none that it was soon followed by others of the same description." Sir Walter was not without sufficient warning, but the long habit of literary labour was too strong for him; and after so distinct a notice of the state of the material organ, he still worked as industriously as ever. During the following winter his state of mind was distressingly shown to his amanuensis. Mr. Lockhart observes,—"A more difficult and delicate task never devolved upon any man's friend, than he had about this time to encounter. He could not watch Scott from hour to hour—above all, he could not write to his dictation—without gradually, slowly, most reluctantly, taking home to his bosom the conviction that the mighty mind, which he had worshipped through more than thirty years of intimacy, had lost something, and was daily losing something more, of its energy. The faculties were there, and each of them was every now and then displaying itself in its full vigour; but the sagacious judgment, the brilliant fancy, the unrivalled memory, were all subject to occasional eclipse.

'Along the chords the fingers stray'd,
And an uncertain warbling made.'

Ever and anon he paused and looked round him, like one half-waking from a dream, mocked with shadows. The sad bewilderment of his gaze showed a momentary consciousness that, like Samson in the lap of the Philistine, 'his strength was passing from him, and he was becoming weak like unto other men.' Then came the strong effort of aroused will—the clouds dispersed as if before an irresistible current of purer air—all was bright and serene as of old, and then it closed again in yet deeper darkness." Under these circumstances it was no wonder that his medical advisers assured him repeatedly and emphatically that if he persisted in working his brain, nothing could prevent his malady from recurring with redoubled severity. His answer was, "As for bidding me not work, Molly might as well put the kettle on the fire, and say, *now don't boil*. . . . . I foresee distinctly, that if I were to be idle, I should go mad!" The fate of Swift and Marlborough were also before his eyes; and in his journal there is an entry expressive of his fear least the anticipated blow should not destroy life, and that he might linger on, a driveller and a show. "I do not think my head is weakened" (this was a subsequent entry)—"yet a strange vacillation makes me suspect. Is it not thus that men begin to fail—becoming, as it were, infirm of purpose?

> "That way madness lies—let me shun that.
> No more of that———."

And when at the court house of Jedburgh he faced the rabble populace and braved their hootings, the same idea of impending calamity was still present to his mind, as he greeted them on turning away, in the words of the doomed gladiator, "*Moriturus vos saluto.*" "As the plough neared the end of the furrow," to use Scott's own expressive phrase, he was still urged on by his fixed habits of labour. "Under the full consciousness that he had sustained three or four strokes of apoplexy or palsy, or both combined, and tortured by various attendant ailments, cramp, rheumatism in half his joints, daily increasing lameness, and now of late gravel, (which was, though last, not least,) he retained all the energy of his will, and struggled manfully against this sea of troubles."

Perhaps there is nothing more remarkable in literary men than this enchantment with labour, and hardly anything less distressing when rest is needed. The mind seems as if it were a wild horse, to which the body is helplessly fastened; or as if it were an imperious tyrant, demanding incessant toil. Hardly is one literary undertaking completed—often before the finishing touches are put to the work—and the "maker" is casting about for another undertaking. This peculiarity in literary men is one of the most obvious, most strongly marked, and most fatal.

Leland was the Sir Walter Scott of his day. Beloved by his king and devoted to the history and antiquities of his country, like Scott, he was a more accomplished scholar; for his ample mind embraced the languages of Greece and Italy, of modern times, and of those out of which English arose. He was a great traveller on the European continent, and he cultivated poetry with ardour. As the "king's antiquary," he spent six years in the survey and study of our national antiquities. He travelled over every county; surveyed towns, cities, and rivers, examined castles, cathedrals, monasteries, tumuli; investigated coins, and copied manuscripts and inscriptions, "yn so muche that," (as he writes, in his 'New Year's Gift to Henry VIII.') "al my other Occupations intermitted, I have so traveled yn yowr Dominions booth by the Se Costes and the midle Partes, sparing nother Labor nor Costes, by the space of these vi Yeres paste, that there is almoste nother Cape, nor Bay, Haven, Creke or Peere, River, or Confluence of Rivers, Breches, Waschis, Lakes, Meres, Fenny Waters, Montaynes, Vallies, Mores, Hethes, Forestes, Chases, Wooddes, Cities, Burges, Castelles, principale Manor Placis, Monasteries, and Colleges, but I have seene them; and notid yn so doing a hole worlde of Thinges very memorable." The vast accumulations of materials which resulted from this industry, occupied him another six years to shape and polish. And his bibliographical were as great as his itinerant labours. He was learned in "Greek, Latin, French, Italian, Spanish, British, Saxon, Welsh, and Scottish" literature. Like Sir Walter Scott, he was an ardent patriot, and the great end and aim of all his toils was the renown of his native land. He trusted so to write its ancient history, that the old glory of renowned Britain should "reflorisch thorough the worlde." But the mighty intellect succumbed to the overwhelming struggle. His conceptions were too great for his frame; so that when about to complete his undertaking he became maniacal, and died in his fortieth year; or, in the words of honest William Burton the antiquary, "Sed cum hoc rude chaos et pergrandis acervus digerendus et in ordinem methodicum redigendus esset, nam vel sui diffidentia non perficiendi hæc magna quæ pollicitus est laborans, vel terrore immensitatis tantæ et tam vastæ molis devictus, confuso et vitiato cerebro è potestate mentis suæ decidit et phrenetica mania (quod lugendum sane) expiravit." The melancholy that cherishes genius may also destroy it, is the sound remark of the author of "The Curiosities of Literature." "Leland brooding over his voluminous labours, seemed to love and to dread them; sometimes to pursue them with rapture, and sometimes to shrink from them with despair." He feared, to use his own language,

> "———— ne pereant brevi vel horâ
> Multarum mihi noctium labores
> Omnes, et patriæ simul decora
> Ornamenta cadant."

*Insanity*, in its various forms, is by no means an unfrequent result of an overworked mind. A painfully interesting illustration is afforded to us by a little episode in Miss Mitford's "Recollections," respecting

Clare, as the insanity was rather that of the imagination than the instincts or feelings. Miss Mitford remarks, "A few years ago he was visited by a friend of mine, who gave me a most interesting account of the then state of his intellect. His delusions were at that time very singular in their character; whatever he read, whatever recurred to him from his former reading, or happened to be mentioned in conversation, became impressed on his mind, as a thing that he had witnessed and acted in. My friend was struck with the narrative of the execution of Charles I., recounted by Clare as a transaction that had occurred yesterday, and of which he was an eye-witness; a narrative the most graphic and minute; with an accuracy as to costume and manners far exceeding what would probably have been at his command if seen. It is such a lucidity as the disciples of Mesmer claim for clairvoyance. Or he would relate the battle of the Nile and the death of Nelson with the same perfect keeping, especially as to seamanship, fancying himself one of the sailors who had been in the action, and dealing out nautical phrases with admirable exactness and accuracy, although it is doubtful if he ever saw the sea in his life."

But, perhaps, of all the ills to which excessive mental labour gives rise, *melancholia* and the *suicidal* monomania are the most distressing. The insane hand has thus stolen away many a valuable life, which might with the most ordinary precaution have been saved. The lamented death of the late Marquis of Londonderry, supervened upon excessive devotion to those toils of state, which, for some few days at least before his death, manifested the ravages they were committing on the organ of intellect. Often the attack is sudden, oftener it is preceded by a predisposition to lowness of spirits, and by thoughts of the most depressing kind. Sir Walter Scott remarks upon this state of feeling when he would have thrown away his life, as a child a broken toy: "Imagination renders us liable to be the victims of occasional low spirits. All belonging to this gifted, as it is called, but often unhappy class, must have felt that but for the dictates of religion, or the natural recoil of the mind from the idea of dissolution, there have been times when they would have been willing to throw away life as a child does a broken toy. I am sure, I know, one who has felt so. O God! What are we?—Lords of nature?—Why, a tile drops from a house-top, which an elephant would not feel more than the fall of a sheet of pastboard, and there lies his lordship. Or something of inconceivably minute origin, the pressure of a bone, or the inflammation of a particle of the brain takes place, and the emblem of the Deity destroys himself or some one else." The narrative of the poet Cowper, in which he describes his mental condition during one of his paroxysms of suicidal melancholia, is as touching as it is instructive. The intolerable anguish—the impulse to self-destruction—the vain struggle to resist, or bravely endure :

> "O wretched state! O bosom! black as death!
> O limèd soul, that, struggling to be free,
> Art more engaged! Help, Angels, make assay!"*

Perhaps amongst the modern victims of overwork who thus perished, Samuel Laman Blanchard merits special notice. His memoir, by Sir Edward Bulwer Lytton, prefixed to his "Sketches from Life," is a touching biographical sketch. "Few men had experienced more to sour them than Laman Blanchard, or had gone more resolutely through the author's hardening ordeal, of narrow circumstance, of daily labour, and of that disappointment in the higher aims of resolution, which must almost inevitably befal those who retain ideal standards of excellence, to be reached but by time and leisure, and who are yet condemned to draw hourly upon unmatured resources for the practical wants of life. To have been engaged from boyhood in such struggles, and to have preserved, undiminished, generous admiration for those more fortunate, and untiring love for his own noble yet thankless calling; and this with a constitution singularly finely strung, and with all the nervous irritability which usually accompanies the indulgence of the imagination, is a proof of the rarest kind of strength, dependent less upon a power purely intellectual, than upon the higher and more beautiful heroism which woman, and such men alone as have the best feelings of a woman's nature, take from instinctive enthusiasm for what is great, and uncalculating faith in what is good." Like Byron, Laman Blanchard had a predisposition to cerebral disorder. At an early age he experienced a paroxysm of suicidal excitement; in the earlier part of his life he abstained wholly from animal food—an undoubted mark of eccentricity to the eye of the physician, whatever vegetarians may say or think; and it was during an acute attack of cerebral irritation that he perished. It was ushered in, however, with the usual warnings. When eking out his income by "a constant waste of intellect and strength," his wife was seized with paralysis and became subject to fits. His vivacity now failed him, and he became subject to deep depression of spirits. "His friends, on calling suddenly at his house, have found him giving way to tears and vehement grief, without apparent cause. In mixed society he would strive to rally; sometimes with success—sometimes utterly in vain. He has been obliged to quit the room to give way to emotions which seemed

* *Hamlet, Prince of Denmark*, act iii., sc. 3.

to arise spontaneously, unexcited by what passed around him, except as it jarred, undetected by others, upon the irritable cords within. In short, the nerves, so long overtasked, were giving way. In the long and gallant struggle with circumstances, the work of toil told when the hour of grief came." Amidst all this, his constant thought was of fresh literary enterprises; a "limèd soul" he was, yet not struggling to be free. So long had he toiled that the image of toil literally dogged him. He chalked out schemes, more numerous, and even more ambitious than any in which he had before indulged. Amongst the rest he meditated "a work upon the boyhood and youth of eminent men;" (we quote his biographer) "on which he wrote to consult me, and for which I ransacked my memory to supply him with anecdotes and illustrations. He passed whole days—even weeks—without stirring abroad, writing and grieving as it were together."

In this short sketch, how clearly the psychiatric practitioner recognises the premonitory symptoms of cerebral congestion—how deeply he grieves that no warning voice was raised—no helping hand stretched forth to snatch him from the abyss, upon the verge of which he evidently stood. The rest followed quickly. Intolerance of light—an attack of hemiplegia—imperfection of vision—spectral illusions—terrible forebodings of some undefined calamity—violent delirium—suicidal impulse—and then the act itself.

We once more quote his biographer—because some apology is due to our readers for this harrowing history—for the moral. "Thus, at the early age of forty-one, broken in mind and body, perished this industrious, versatile, and distinguished Man of Letters. And if excuse be needful for dwelling so long upon details of a painful nature, it may be found in the deep interest which science takes in the pathology of such sufferers, and in the warnings they may suggest to the labourers of the brain when the first ominous symptoms of over-toil come on, and while yet repose is not prescribed too late."

Laman Blanchard was the biographer of a kindred sufferer—L. E. L. *Her* history, also, is not without an emphatic warning; but we forbear to dwell longer upon this painful subject. There is one other result of mental labour which, however, deserves notice—namely, that in which the horrors of confirmed *hypochondriasis* afflict the toiler. This shows itself, not merely in the common form of weak fancies as to the bodily health, or in unaccountable gloom, but also in a less understood form, in which the judgment is weakened, and the individual gets committed to some intellectual folly in science or literature, religion or politics. The man is not actually insane, or, if insane, there is method in his madness; but his feelings are easily acted upon, his credulity is unbounded, and his actions consequently unworthy his reputation or his intellect. We feel that this is delicate ground, and we therefore avoid specifying particular instances, not desiring to hurt conscientious convictions, whether in science or religion, although they are only held and expressed after (as we think) the mind is weakened by overwork. We may, however, quote here a medical review of high authority, without risk of offence. The remarks are made in reference to the disease termed "cerebropathy" by some, by others "nervousness," and by others "brain-fag," treated very successfully by certain empirics:—"A disease of literary, political, and professional men—of men who have changed night into day, either in the pursuit of science, literature, or pleasure, and robbed the brain of the repose necessary to its vigorous action. In such, a hypochondriacal condition verging upon insanity is the real state: the brain is enfeebled, the mind is in a degree imbecile, the imagination predominant. It is with this disease upon them, that men of refinement, of genius, of learning, of high station in their respective walks, fall a prey to quacks, religious and medical, and become the subjects of homœopathic, hydriatic, and mesmeric treatment; or, still worse, abandon friends and the healthy useful employments of vigorous manhood, for the pursuit of ecclesiastical phantoms or the rigour of an ascetic 'retreat.'"

Although we have hitherto illustrated the history of the overworked mind by examples drawn from literature, we do not by any means wish it to be understood that it is peculiar to this class of intellectual toilers. The bar, the parliament, the exchange, the universities, and the numerous minor channels in which energetic mental labour predominates, all supply ample illustrations. Still, in the literary class of men we have presented to us the *type* of the whole, and whatever is applicable to them is applicable also to the others. There are varieties, however, determined more or less by the sedentary, or gregarious, or active habits, of the individual; and there is one important class of overworkers, in whom the brain is worked before it has attained its full development and capacity for labour. This class includes *young* persons of all kinds, to whom academical emulation, or the *res angustæ domi*, acts as a stimulus to excessive mental toil.

Perhaps the *overworked student* is as familiar an instance of the fearful results which follow on excessive mental culture, as the overworked literary man. The universities and colleges afford numerous examples, and it is somewhat difficult to select one from the number. It is of importance to remember that the glaring instances (such as that of Henry Kirke White) are not the most instructive or the most frequent. For one victim who sinks down in the heat of the battle, amidst the sympathies of an admiring public, two or three are doomed to a life of dull mediocrity or intellectual imbecility. The violent effort may not have induced insanity, or any obvious disease of the intellect, yet from the time that it was accomplished, the student ceases to labour as was his wont, and the early promise of talent and usefulness is effectually defeated. It was the fate of Southey to suffer at the close of his career from the same cause which arrested the course of the two brother poets whose sufferings he related, namely, Chatterton and Kirke White. Chatterton was an illustration of the indigent *littérateur* perishing by his own hand, White of the student ambitious for academical honours, perishing at the moment of victory. White overworked himself before he went to Cambridge, and had doubtless thereby enfeebled a cerebral fibre never strong. While still an articled clerk at the age of eighteen, we are informed by his biographer that after the ordinary duties of the day, he "allowed himself no time for relaxation, little for his meals, and scarcely any for sleep. He would read till one, two, three o'clock in the morning; then throw himself on the bed, and rise again to his work at five, at the call of a *larum*, which he had fixed to a Dutch clock in his chamber. Many nights he never lay down at all. It was in vain that his mother used every possible means to dissuade him from this destructive application." His health soon sunk under these habits; and his constitution experienced a shock which it never recovered. During his first term at Cambridge he had to try for a university scholarship, as well as to pass the general examination. "Once more he exerted himself [for the latter] beyond what his shattered health would bear, and he went to his tutor, Mr. Catton, with tears in his eyes, and told him that he could not go into the hall to be examined. Mr. Catton, however, thought his success here of so much importance, that he exhorted him with all possible earnestness to hold out the six days of the examination. Strong medicines were given him, to enable him to support it; and he was pronounced the first man of his year. But life was the price which he was to pay for such honours as this: and Henry is not the first young man to whom such honours have proved fatal. He said to his intimate friend, almost the last time he saw him, that were he to paint a picture of Fame crowning a distinguished under-graduate after the senate-house examination, he *would represent her as concealing a death's head under a mask of beauty*." In his letters, Kirke White gives sad glimpses of the state of his mind while at Cambridge. He was overwhelmed, previously to his examination, with melancholy. "I wandered up and down," he writes at the close of 1805, "from one man's room to another, and from one college to another, imploring society, a little conversation, and a little relief of the burthen which pressed upon my spirits." In February following (1806) he says, "The state of my health is really miserable; I am well and lively in the morning, and overwhelmed with nervous horrors in the evening. I do not know how to proceed with regard to my studies—a very slight overstretch of the mind in the day-time occasions me not only a sleepless night, but a night of *gloom* and horror. The systole and diastole of my heart seem to be playing at ball—the stake my life." How significant these premonitory phenomena—how vivid the warning to him who could read them aright. The next stage (of congestion) our readers will be prepared for. "Last Saturday morning" (we quote again from one of his letters, dated July, 1806) "I rose early, and got up some rather abstruse problems in mechanics for my tutor, spent an hour with him, between eight and nine, got my breakfast, and read the Greek history (*at breakfast*) till ten, then sat down to decipher some logarithm tables. I think I had not done anything at them when I lost myself. At a quarter past eleven my laundress found me bleeding in four different places in my face and head, and insensible. I got up and staggered about the room, and she, being frightened, ran away and told my Gyp to fetch a surgeon. Before he came, I was sallying out with my flannel gown on, and my academical gown over it," &c. A few weeks after this he went to London to relax—"the worst place," as Southey very correctly remarks, "to which he could have gone; the variety of stimulating objects there hurried and agitated him, and when he returned to college he was so completely ill that no power of medicine could save him. His mind was worn out; and it was the opinion of his medical attendant, that if he had recovered, his intellect would have been affected." He first became delirious, then sunk into stupor, and so died. How pregnant a warning is this history to ambitious tutors and parents! What a lesson against aiming for "the bubble reputation" instead of a fitness for solid usefulness through a prolonged life. A sad disappointment, indeed, it is,—to quote White's own lines,—

"———— to find,
When life itself is sinking in the strife,
'Tis but an airy bubble and a cheat!"

Having so fully illustrated the consequences of unnatural toil of the mind, it is incumbent on us to point out the remedy. This has been long understood, and is obvious. In one word, it is REST. It is the removal of the cause—the first step in the cure of all diseases. But it is not so easy to apply this remedy to the special cases under consideration, partly because in by far the larger proportion the toil is almost imperatively demanded by circumstances, partly because, as we have seen, the habit for labour of the kind has so fixed itself, that it is all but irresistible. It is of far greater importance that the labourer shall so labour that he shall gather strength, and not weakness, from his toil, in accordance with the order of Divine Providence. To this end there is only one way, namely, to labour in humble subjection to the laws of our mental and corporeal well-being. Intellectual labour need not necessarily induce the frightful ills we have described or catalogued; on the contrary, it is that by which the progressive development of mankind as a created being can alone be secured. It is therefore not merely the privilege, but the duty of every man to work his intellectual faculties to the utmost limit consistent with sound health, so that he may thereby not only add to the general stock of wisdom and knowledge, but also so act upon himself corporeally, that some part of that improvement in his mental powers with which mental labour rewards him, may be transmitted to a vigorous offspring.

In analysing the histories of many victims to intellectual toil, we cannot but be struck with the general fact, that a total disregard of their *bodily* health was as much a moving cause of their disasters as their prolonged mental efforts. The man who neglects the ordinary appliances of health, and the ordinary rules of existence, cannot fail to suffer. Nervousness, and melancholy, and low spirits, are as much the lot of the luxurious, the indolent, and the dissipated, as of the man of letters, the statesman, or the merchant. The prevention of the morbid results we have alluded to is comprised in the word SELF-DENIAL. A voluminous writer of the last century lived to be 87 years of age. He not only was a great commentator, a philosopher, an encyclopædist, a divine, but he had upon his mind the care of the whole body of "the people called Methodists," and who now bear his name. It was only by his sound common sense, his self-denial, and his sense of duty, that he was enabled to be "in labour more abundant." As an amusing instance of John Wesley's practical common sense, we extract the following from his advice to his preachers, whom he ruled as a preceptor as well as a father. Some of them were complaining at a "Conference" held at Leeds in the year 1778, of being "nervous," and suffering from nervous disorders. As to these, he observes, (we quote from the published minutes)—

"Q. What advice would you give to those that are nervous?

"A. Advice is made for them that will take it. But who are they? One in ten, or twenty?

"Then I advise:—

"1. Touch no dram, tea, tobacco, or snuff.

"2. Eat very light, if any supper.
"3. Breakfast on nettle or orange-peel tea.
"4. Lie down before ten;—rise before six.
"5. Every day use as much exercise as you can bear; or,
"6. MURDER YOURSELF BY INCHES!"*

We do not know that much can be added to this quaint but sound advice. Daily exercise, early rising, the total abnegation of spirits, fermented drinks, tobacco in any form, and tea, dinner in the middle of the day, are rules which any intelligent man must see are particularly applicable to those who work the nervous system exclusively. Daily exercise must be taken to balance cerebral with muscular activity. Stimulants to the nervous system must be avoided, because it is already over-stimulated by thought. Repose for the brain and sensorial nerves, must be secured by going early to rest, because nature has ordained that repose is necessary for their healthy action, and because the hours of darkness after sunset, are universally the hours of repose of those animals that are not nocturnal in their habits. Abstinence from gross living is requisite, because the waste of the system is not in the muscles but in the *minor* agent, as regards material extent—the cerebrum.

It is, perhaps, as to the mode in which these habits can be practised that there will be the greatest difference of opinion. It is very easy to prescribe daily exercise to the hard-working statesman, or man of letters, or professional man, but how is he to secure it amidst the hurry of metropolitan life, and in the wilderness of baked clay and granite of metropolitan streets? Early to rest may be most wholesome, but how is it practicable with the present arrangements of daily life in the larger towns? Strong tea may be "bad for the nerves," but without it the jaded student truly says, "I should have no nerves at all! and as for avoiding tobacco, how could I exist without my delicious Havannah, the sole solace of my studies?" Thus, secondary circumstances, as well as the primary necessity, bind the intellectual labourer to a wearisome health-destroying cycle of influences to which he is helplessly subject, and from which it is only by efforts, almost superhuman, that he can escape.

The prevention of disease under circumstances like these, can only be attained by a united effort and a combination of all those interested. Thus made it is not surely quite an impossibility. The stimulus of emulation might excite to athletic exercises; and steady advocacy through the press of more rational hours for social enjoyment, might do much in modifying the late hours of fashionable life; an earlier dinner

* Minutes of the Methodist Conference. Ed. 1812. Vol. i. p. 136.

hour, morning operas, &c., would not be altogether useless. It is, however, quite in the power of the individual to do much for himself. Thorough ablution of the head once or twice a-day with cold water, or even a slight shower bath, will do much service to the material organ. Extreme temperance in diet would also keep the head clear; but, above all, cessation from mental effort, so soon as the premonitory symptoms of over-work show themselves. Hot eyes, flushed face, irritable temper, despondency, uneasy slumbers, slight vertigo, or, during sleep something like somnambulism instead of dreams, should be attended to instantly. If any of these supervene, a cessation from labour is strenuously indicated. From that moment, all head-work is out of the capital stock of strength; it is true wear and tear, and the loss thus incurred must either be speedily replaced, or disorder and disease will result. Physiological laws, it cannot be too well remembered, are as inexorable as the physical. The rest is comprised in two things; —GENTLE BODILY EXERCISE, SLEEP.

No man who works his brain actively should work all the year round. Of all organs of the body it is that which the most enjoys a holiday. The most practicable and the most useful is a pedestrian excursion, and upon this point we would again quote from the "British and Foreign Medico-Chirurgical Review." "In this class of cases there is a more legitimate remedy than these empirical [the hydropathic] appliances, and that is, a pedestrian tour, such as Dr. Forbes enjoyed, and has described in his pleasant 'Physician's Holiday.' Let the man of refinement and imagination, who is pestered with thick-coming fancies, especially after reading 'The Fathers,' and feels that he has lost the healthy, noble feeling of self-reliance, which characterises the true man, flee to the mountains for solace, rather than to an ascetic, enthusiastic priest. Let him defer the performance of what he thinks to be a duty, and the practice of what he yearns for, as a refuge from his gloom, until he has strengthened *the organ of thought*, and enjoys a *mens sana in corpore sano.* Without this, his sacrifices and martyrdom are but the self-imposed evils of a foolish hypochondriac, and of no religious value whatever. If, after breaking away from all his engrossing studies, and holding converse with nature in her sublimest aspects—drinking nothing more potent than water—walking twenty miles a-day, and every evening taking a warm bath—if, after a three months' pedestrian tour in the Tyrol, Switzerland, or Scotland, so conducted, he returns to the world and finds its aspect towards him unchanged, and he has no desire to do his duty—*solid* duties—actively and earnestly, then there is nothing for him but to 'retreat,' and live amidst the phantoms and chimeras which are to his taste. 'Hellebore'

will not cure him; Bath, the Brünnen, and Malvern will be alike useless; and even the false miracles of Mesmerism will 'pale their ineffectual ray,' before those of another class, which to his morbid imagination appear real."*

There is still another class of head-workers—those to whom no holiday comes, to whom a pedestrian excursion is too great a luxury to be even dreamed of, and who *must* work at all hazards. These may ward off many evils by a strict diet and regimen, and by *varying from time to time the subject of their studies.* This is the great secret of *safe* continued head-work. It is a species of cerebral gymnastics, by which all parts of the organ of thought are equally worked. With this and a sedulous attention to the bodily health, by the simple means which common sense dictates, many have been enabled to work long and strenuously with comparative impunity; and, although the evil day must come at last, it is long deferred.

We have offered to the man of mind few other than what may appear selfish motives to induce him to guard well the powers God has given him. We have not forgotten, however, that from him to whom much is given much also will be required. Unless this higher motive of duty direct the labourer in the field of intellect; unless he guard his gifts as things held only in trust, and uses them as one who must render an account—he will spend his days in labour, and late take rest in vain. Too late he will learn by bitter experience that, in his case,

"Life's but a walking shadow; a poor player,
That struts and frets his hour upon the stage
And then is heard no more: it is a tale
Told by an idiot, full of sound and fury,
Signifying nothing."

* Opere Citato. Vol. vii. p. 452.

